# *In vivo* 3D imaging of ovarian cancer outgrowth in transgenic mouse model with optical coherence tomography

**DOI:** 10.1117/1.JBO.30.9.096007

**Published:** 2025-09-30

**Authors:** Huan Han, Aleese Mukhamedjanova, Denise C. Connolly, Marcin P. Iwanicki, Shang Wang

**Affiliations:** a Stevens Institute of Technology, Department of Biomedical Engineering, Hoboken, New Jersey, United States; b Fox Chase Cancer Center, Philadelphia, Pennsylvania, United States; c Stevens Institute of Technology, Department of Chemistry and Chemical Biology, Hoboken, New Jersey, United States

**Keywords:** optical coherence tomography, ovarian cancer, tumor outgrowth, intravital window, 3D imaging, transgenic mouse model

## Abstract

**Significance:**

Peritoneal dissemination is the major mechanism of how ovarian cancer (OC) spreads. It features tumor outgrowths in the form of multicellular spheroids, their detachment from the primary site, and their implantation in the peritoneal cavity. To understand this process, analyzing the outgrowths at their native locations within the female reproductive system is essential. However, *in vivo* study of the OC outgrowths remains unattainable primarily due to the lack of *in vivo* imaging approaches to probe such small tumor structures at a high resolution.

**Aim:**

We address this technical challenge by establishing *in vivo* high-resolution 3D imaging of the OC outgrowths in the mouse model.

**Approach:**

This *in vivo* imaging approach relies on optical coherence tomography (OCT) for 3D label-free imaging and an intravital window to bypass the mouse skin and muscle layers. To demonstrate the imaging capability, we use Tg*MISIIR*-*TAg* transgenic mice that develop spontaneous epithelial OC. The normalized surface lengths of the ovary and the OC outgrowth are measured from OCT images to characterize the tissue morphology. Immunohistochemistry staining is employed to confirm the presence of transgene-positive cells in OC and outgrowths.

**Results:**

We present the first *in vivo* high-resolution 3D image of the OC outgrowths in the mouse model. The tissue morphology and structure of OC outgrowths have striking differences from the normal ovary, which is quantitatively assessed and compared. We further show that OC outgrowths within and growing out of the ovarian bursa, revealing the difference in their surface morphologies. We also present the detached OC outgrowths and the fluid-filled chambers inside OC, both with 3D quantifications showing the heterogeneity of their volumes.

**Conclusions:**

This *in vivo* OCT imaging approach in the mouse model enables high-resolution assessment of detailed 3D structures of OC outgrowths, paving the way for *in vivo* study of the OC dissemination process.

## 1 Introduction

Ovarian cancer (OC) is the deadliest gynecological cancer.^1^ Epithelial OC accounts for over 95% of OC cases^2^ and possesses a unique spreading process.^3^ Unlike other malignancies that metastasize hematogeneously, OC spreads primarily through peritoneal dissemination, which features tumor outgrowths in the form of multicellular spheroids, their detachment from the primary site, and their subsequent implantation into the mesothelium in the peritoneal cavity.^4^ A fundamental understanding of this entire process is key to improving the clinical management of epithelial OC, which has seen a ∼50% 5-year survival rate that has only moderately changed over decades.^5^ Although the survival and implantation of the already detached OC outgrowths can naturally be studied *in vitro* by collecting them from the ascites (the excess fluid in the peritoneal cavity),^6^^–^^8^ understanding the formation and detachment processes of the OC outgrowths requires studies at their native locations on the ovary, given the complex, dynamically varying environment of the female reproductive system. However, such studies are challenging primarily due to the lack of *in vivo* imaging approaches that can probe the OC outgrowths in the animal model.

As a multicellular spheroid, the OC outgrowth is on the scale of tens to hundreds of microns. *In vivo* imaging of OC in the mouse model has been demonstrated with a few imaging modalities and approaches. Near-infrared fluorescence imaging and magnetic resonance imaging are frequently used to evaluate the tumor size and spread of OC,^9^^–^^11^ but their relatively low resolution cannot resolve the outgrowths. Multiphoton microscopy of OC through intravital window, surgery, or laparoscopy can provide high-resolution imaging of the individual cancer cells and collagen fibers and also generate quantitative analyses;^12^^–^^15^ however, the focus on the cellular level with a limited field of view does not capture tumor outgrowths. Fluorescence microendoscopy has demonstrated high-quality, microscopic imaging of disseminated nodules (spheroids) in the peritoneal cavity^16^ but is not capable of 3D imaging. As such, considering the imaging scale, modalities that provide tissue-level 3D imaging with a microscale resolution are ideal for assessment of the OC outgrowths.

Optical coherence tomography (OCT)^17^ has this required imaging scale. Employing broadband near-infrared light and relying on low-coherence interferometry to resolve depth, OCT features a spatial resolution of ∼1 to 15  μm with a millimeter-level 3D field of view,^18^ suitable for the size of OC outgrowths. In contrast to fluorescence-based imaging, OCT forms its basic structural contrast with the endogenous backscattering of light inside the tissue, thus not requiring exogenous labels. In addition to structural imaging, functional OCT imaging techniques, such as dynamic OCT, are widely available to extract various information at the tissue and cell levels with or without exogenous labels.^19^^–^^27^ This allows OCT to potentially provide registered multicontrast imaging, linking tissue structures of the OC outgrowths with their biophysical properties. *In vitro* and *ex vivo* OCT structural and functional imaging of the mouse ovary have been previously reported, showing the applicability of OCT for analyzing tissue structures and follicles in the ovary.^28^^–^^30^ In particular, texture analysis and convolutional neural networks have been utilized with the OCT structural image of *ex vivo* ovary samples for OC detection,^31^^,^^32^ indicating the feasibility of distinguishing cancerous ovarian tissues based on the OCT structural imaging contrast. *In vivo* OCT imaging of the mouse ovary was also demonstrated through a surgery that opens the body cavity and exposes the reproductive organs.^33^ Notably, through a similar surgical approach, *in vivo* OCT imaging of OC in the mouse model was performed as well,^15^ but 3D imaging of the OC outgrowths has not been reported.

Here, we report the first *in vivo* 3D imaging of the OC outgrowths in the mouse model using OCT and an intravital window. With Tg*MISIIR*-*TAg* transgenic mice that develop spontaneous epithelial OC, we show detailed 3D structures of OC and its outgrowths, verified by immunohistochemistry (IHC) and revealing striking morphological differences from the normal ovary in wild-type mice. Our quantitative analysis using the parameter of normalized surface length (NSL) indicates the statistical significance of this difference. We further show OC outgrowths within and growing out of the ovarian bursa, revealing and assessing their different surface morphologies. We also show 3D imaging of the detached OC outgrowths and the quantification of their volumes, suggesting the potential for analyzing the process of outgrowth detachment. Finally, we present 3D imaging of fluid-filled chambers within OC, indicating their heterogeneity in size through volume measurements. This work sets the stage for high-resolution analysis of OC dissemination *in vivo* in the mouse model, opening the door to linking molecular markers and treatment responses with the outgrowth formation and detachment at the native site of the OC development and progression.

## 2 Materials and Methods

### 2.1 OCT System

We used a spectral domain OCT system for this study. The details of the system were presented in our previous work.^34^ Briefly, the system employed a supercontinuum laser (SuperK EXTREME EXR-9 OCT, NKT Photonics, Birkerød, Denmark), and through filtering, we utilized a wavelength range of 779 to 925 nm that were mapped to the 2048 pixels of a line-scan camera (OctoPlus, Teledyne e2v) with an A-scan rate of up to 250 kHz. A scan lens (LSM03-BB, Thorlabs, Newton, New Jersey, United States) was used for laser scanning through a set of galvanometer mirrors. We had an axial resolution of ∼6  μm in air and a transverse resolution of ∼9  μm, with an available depth range of ∼2.5  mm and a transverse field of view of ∼8  mm by ∼8  mm. For 3D imaging of OC and its outgrowths in the mouse model, we used an A-scan rate of 100 kHz with each volume taking 18 s to acquire. The transverse spatial sampling was maintained at ∼3  μm in both directions, ensuring a high quality of the OCT image.

### 2.2 Transgenic Mouse Model

**Fig. 1:**
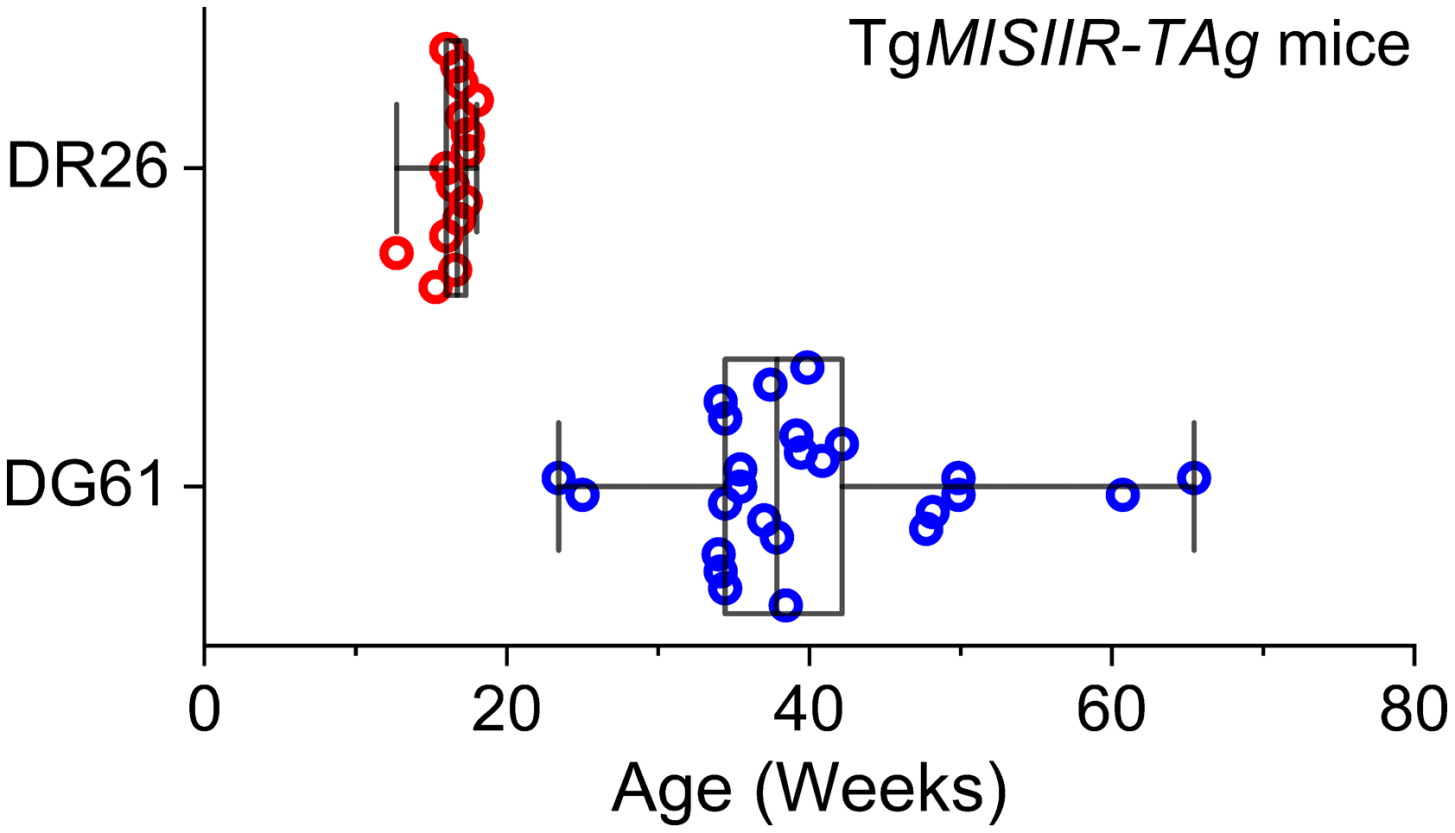
Ages of the Tg*MISIIR*-*TAg*-*DR26* mice and the Tg*MISIIR*-*TAg-DG61* mice used in this study. Box plots with median line and all data points. Whiskers are the minimum and maximum.

All animal procedures were approved by the Institutional Animal Care and Use Committee at Stevens Institute of Technology, and all experiments followed the approved guidelines and regulations. The Tg*MISIIR*-*TAg* transgenic mouse model with C57BL/6 background was created by Connolly et al.,^35^ and two Tg*MISIIR*-*TAg* mouse lines were transferred to and maintained at Stevens Institute of Technology for all the imaging experiments. By expressing T-antigen (*TAg*) genes from the Simian virus 40 (SV40) under the Müllerian inhibiting substance type II receptor (*MISIIR*) promoter, the female Tg*MISIIR*-*TAg* mice develop spontaneous bilateral OC.^35^ This transgenic mouse model was the first one developed for epithelial OC,^36^ has been characterized over the years through multiple approaches,^9^^,^^10^^,^^37^^–^^39^ and is a critical resource for studying the OC dissemination. With the expression of the same transgene, the two Tg*MISIIR*-*TAg* mouse lines, named Tg*MISIIR*-*TAg*-*DR26* and Tg*MISIIR*-*TAg-DG61* (referred to as DR26 and DG61, respectively, in this work), have different phenotypes and were both involved in this work. A total of 49 female mice from these two mouse lines have been imaged *in vivo* with OCT for this study, including 15 DR26 mice, 26 DG61 mice, and 9 wild-type mice. Genotyping with PCR for the *TAg* gene was performed to confirm the genotype of each mouse. There has been evidence suggesting that in humans, the high-grade serous ovarian cancer originates in the oviduct (fallopian tube).^40^^,^^41^ Similar to this, in both of the mouse lines, early neoplastic lesions develop in the oviduct; in comparison to the DG61 mice, the DR26 mice more likely have the tumors spread to the ovary, and the spread is relatively faster. Targeting the OC and its outgrowths, we thus used distinct age groups for DR26 and DG61 mice. The age of every Tg*MISIIR*-*TAg* mouse is shown in Fig. 1. In particular, the ages of the DR26 mice we used were 16.4±1.2 weeks, and the ages of the DG61 mice we used were 39.9±9.5 weeks.

### 2.3 *In Vivo* Imaging Setup

**Fig. 2:**
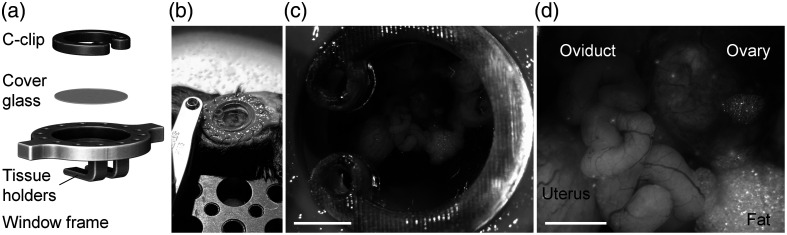
*In vivo* setup with an intravital window implanted on the mouse for OCT imaging of the OC outgrowths. (a) Illustration of the intravital window setup. (b) *In vivo* imaging setup with the intravital window implanted on the mouse under anesthesia on a 37°C platform. *In vivo* bright-field images showing (c) the reproductive organs through the implanted window and (d) the higher-magnification view of the ovary and the oviduct. Scale bars are 3 mm in (c) and 1 mm in (d).

To gain optical access to the mouse ovary, we used an intravital window designed to be implanted on the right dorsal side of the mouse. The window represents a modified version of the one previously reported,^42^ which was originally inspired by Bochner et al.^13^ Specifically, the depth of the window (potential space inside the window) has been increased to accommodate the larger tissue size due to the OC. The window setup is shown in Fig. 2(a), which consists of a window frame, a circular cover glass, and a C-clip. The window frame and the C-clip can be 3D printed at a low cost using the Form 3 stereolithography printer (Formlabs, Somerville, Massachusetts, United States), and both parts are autoclavable. The window frame has 14 eyelets for suturing, two extensions for clamping, and two tissue holders to hold the reproductive organs. The circular cover glass (12-545-81, Fisher Scientific, Hampton, New Hampshire, United States) is made of borosilicate glass with a thickness of 0.16 to 0.19 mm, well suitable for imaging with near-infrared light. For window implantation, the mouse was kept on a 37°C platform and was under anesthesia with isoflurane. A circular piece of skin with a diameter of ∼12  mm above the right ovary was first removed, and the window was sutured to the edge of the skin. A small 2 to 3 mm incision was then made on the muscle layer, and the set of reproductive organs, including the ovary, the oviduct, and part of the uterus, were gently pulled out of the body cavity. Blunt forceps were used to hold only the fat pad associated with the ovary. A drop of tissue adhesive was applied between the tissue holders and the fat pad to secure the ovary onto the tissue holders. The space inside the window was then filled with 37°C 1× phosphate-buffered saline (PBS), and the circular cover glass was used to close the window, secured by the C-clip. Although this study did not involve longitudinal imaging, the window implantation allows for it, with a maximum duration of 7 days, which has been previously tested. The mouse with the window implanted can move freely and has normal feeding and grooming behaviors.^43^

During imaging, the mouse was placed on a 37°C platform and maintained under isoflurane anesthesia. A stabilized clamp was used to hold the window and slightly lift it to minimize the influence of body motion caused by the mouse breathing. A picture of this *in vivo* setup is shown in Fig. 2(b). With a 25 mm working distance from the OCT scan lens, this *in vivo* setup can be conveniently placed under the sample arm of the OCT system for imaging. Figure 2(c) provides a bright-field image of the reproductive organs through the implanted window *in vivo*, with a higher-magnification view in Fig. 2(d), showing the ovary, the oviduct, a small portion of the uterus, and the fat connected to the ovary.

### 2.4 Immunohistochemistry (IHC) Staining

After *in vivo* imaging, the mouse was euthanized, and the ovary with the oviduct and fat pad was removed from the mouse, fixed in formalin for 24 h, and stored in ethanol. The region of OCT imaging was carefully marked with black ink on the sample. The tissue was processed and embedded in paraffin, and sectioning was later performed within the imaged regions to produce sections of a 5  μm thickness, which were then mounted on glass slides. IHC staining for SV40 TAg was performed to identify transgene-positive cells. The sections were deparaffinized using xylene and rehydrated in a series of ethanol solutions and deionized water. The SV40 TAg was retrieved by treating the sections in 1× sodium citrate antigen retrieval solution. BLOXALL endogenous enzyme blocking solution was used to block endogenous peroxidase in the sections and rinsed with 1× PBS. The cellular membranes were then permeabilized using 0.5% Triton X-100 in 1× PBS, followed by two 1× PBS rinses. Sections were incubated at room temperature in a humid chamber for 1 h in 2.5% normal horse serum, followed by an overnight incubation at 4°C in a humid chamber in a 1:100 dilution of mouse anti-SV40 TAg monoclonal antibody (Pab 101) to 2.5% normal horse serum. The next day, slides were rinsed in repeated washes of 1× PBS with 0.1% Tween 20 (PBST). Sections were then incubated at room temperature in a humid chamber for 1 h with biotinylated horse anti-mouse/rabbit immunoglobulins, which were then rinsed with PBST washes. Sections were then incubated in avidin-biotin complex for 30 min and rinsed with PBST washes. Diaminobenzidine was added until a brown color developed and rinsed with deionized water. Counterstaining was performed using hematoxylin and rinsed with water, and coverslips were then mounted on slides using an aqueous mounting media. The slides were imaged using a Zeiss Primovert microscope with a 20× Zeiss objective and with a 4K color camera (Axiocam 208, Zeiss, Jena, Germany) through a 0.5× adaptor. All cell nuclei appear purple with the hematoxylin stain, whereas the IHC staining of TAg appears brown in nuclei, indicating transgene-positive cells. Each IHC image was set with its dynamic range exactly covering the minimum and maximum of its histogram.

### 2.5 Image-Based Assessment of OC Outgrowths

**Fig. 3:**
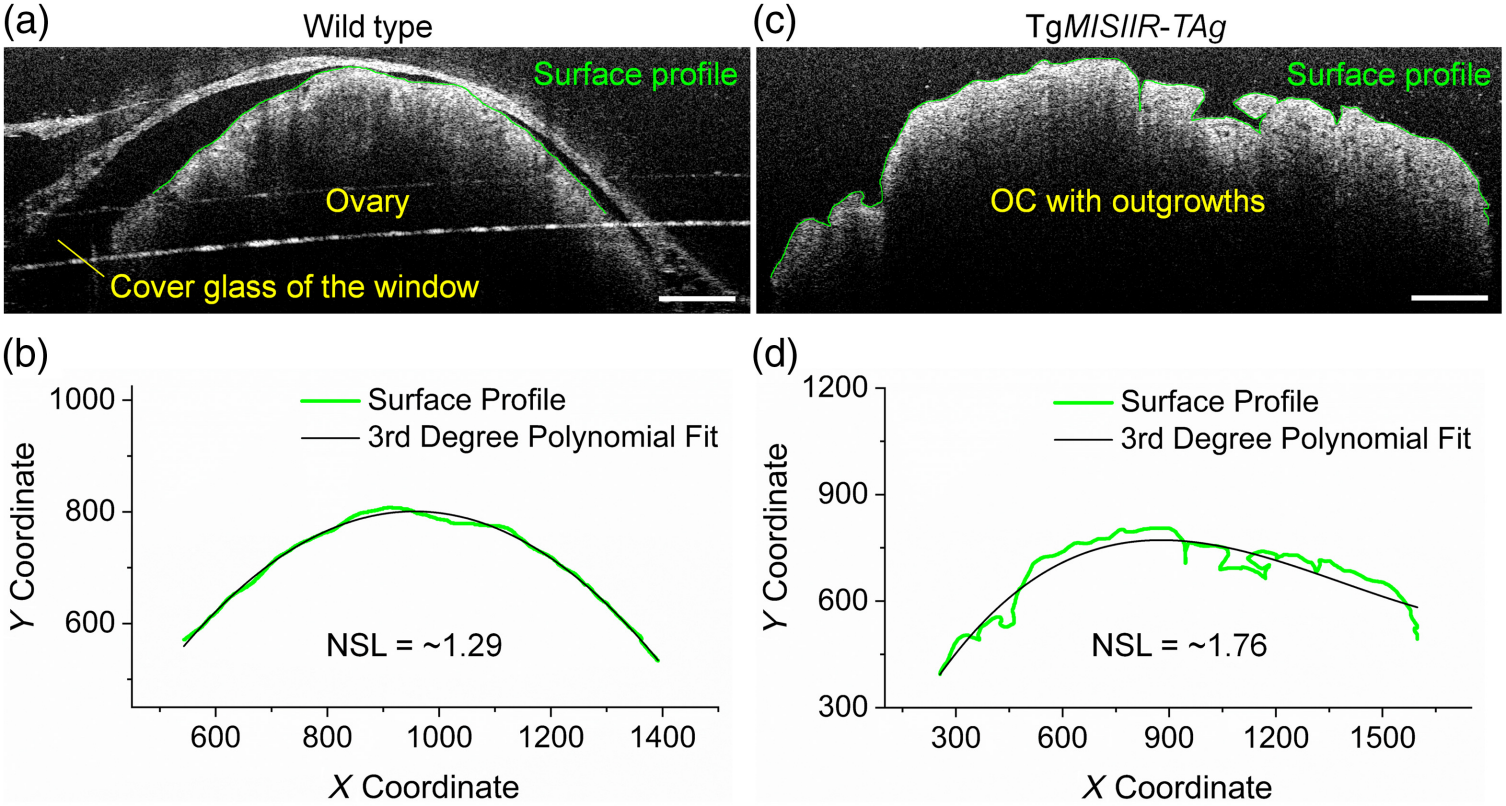
*In vivo* measurement of the NSL of the ovary. Representative B-scan images from (a) a normal ovary of the wild-type mouse and (c) the OC with outgrowths of the Tg*MISIIR-TAg* transgenic mouse, where the surface profiles are marked with green lines. Plots of the surface profiles from the (b) normal ovary and (d) the OC with outgrowths obtained from (a) and (c), respectively, with the corresponding third degree polynomial fits. The ratio of the surface profile length to the polynomial length is defined as the NSL used to assess the OC outgrowths. Scale bars are 300  μm.

To characterize the OC outgrowths, we measured the NSL based on the B-scan OCT images (Fig. 3). The NSL was inspired by the gyrification index^44^ that measures the cortical folding of the brain. Similarly, the NSL measures how complex the ovarian surface profile is, i.e., an overall smoother ovarian surface has a lower NSL, whereas an ovarian surface with extensions and folds has a relatively higher NSL (Fig. 3). Specifically, with a B-scan image, the surface of the ovary (or the tumor with outgrowths) was delineated manually in Fiji,^45^ with two examples shown in Figs. 3(a) and 3(c). The x and y coordinates of the surface profile were obtained and were then fitted with a third-degree polynomial in MATLAB (MathWorks, Natick, Massachusetts, United States), with examples shown in Figs. 3(b) and 3(d). The lengths of the surface profile and the polynomial were measured as $L_{s}$ and $L_{p}$, respectively, and the NSL was calculated as $L_{s}/L_{p}$. For the normal ovaries from wild-type mice, we measured the NSL from five equally spaced B-scan locations, and the values were averaged to represent the mouse. For the OC with outgrowths on the ovaries from Tg*MISIIR*-*TAg* mice, we focused on the OC outgrowth and measured the NSL from selected B-scan images that contain the OC outgrowth. The measured values, if multiple, from each mouse were also averaged to represent the mouse. For measurements of the specific outgrowths that grew out of the bursa, only the NSL values from the B-scans containing those outgrowths were averaged to represent them.

### 2.6 OCT Image Visualization and Processing

Structural images of the ovary, the OC, and its outgrowths were reconstructed in 3D with the OCT signal intensity. Visualizations of the volumes were conducted with Open Chrono-Morph Viewer (OCMV),^46^ and the clipping spline^47^ in OCMV was used to generate nonplanar cutaway views to reveal the structures inside the volume. Both Imaris (version 9.6.0, Oxford Instruments, Abingdon, United Kingdom) with its surface function (surfaces were created with the absolute intensity threshold and a smoothness level of 5  μm) and Fiji^45^ with the manual region-of-interest selection and measurement tool were used to measure the volumes of the detached OC outgrowths and the fluid-filled chambers inside the tumor. The measured volumes are directly reported in the results.

### 2.7 Statistical Analysis

All measurement data are shown with the box plot, and all the data points are displayed. When a comparison between groups is needed, the data in each group were first tested for normality with the Kolmogorov–Smirnov test (α=0.05). In this work, none of the groups had normally distributed data, and thus, all comparisons were through the Wilcoxon rank sum test (α=0.05).

## 3 Results

**Fig. 4:**
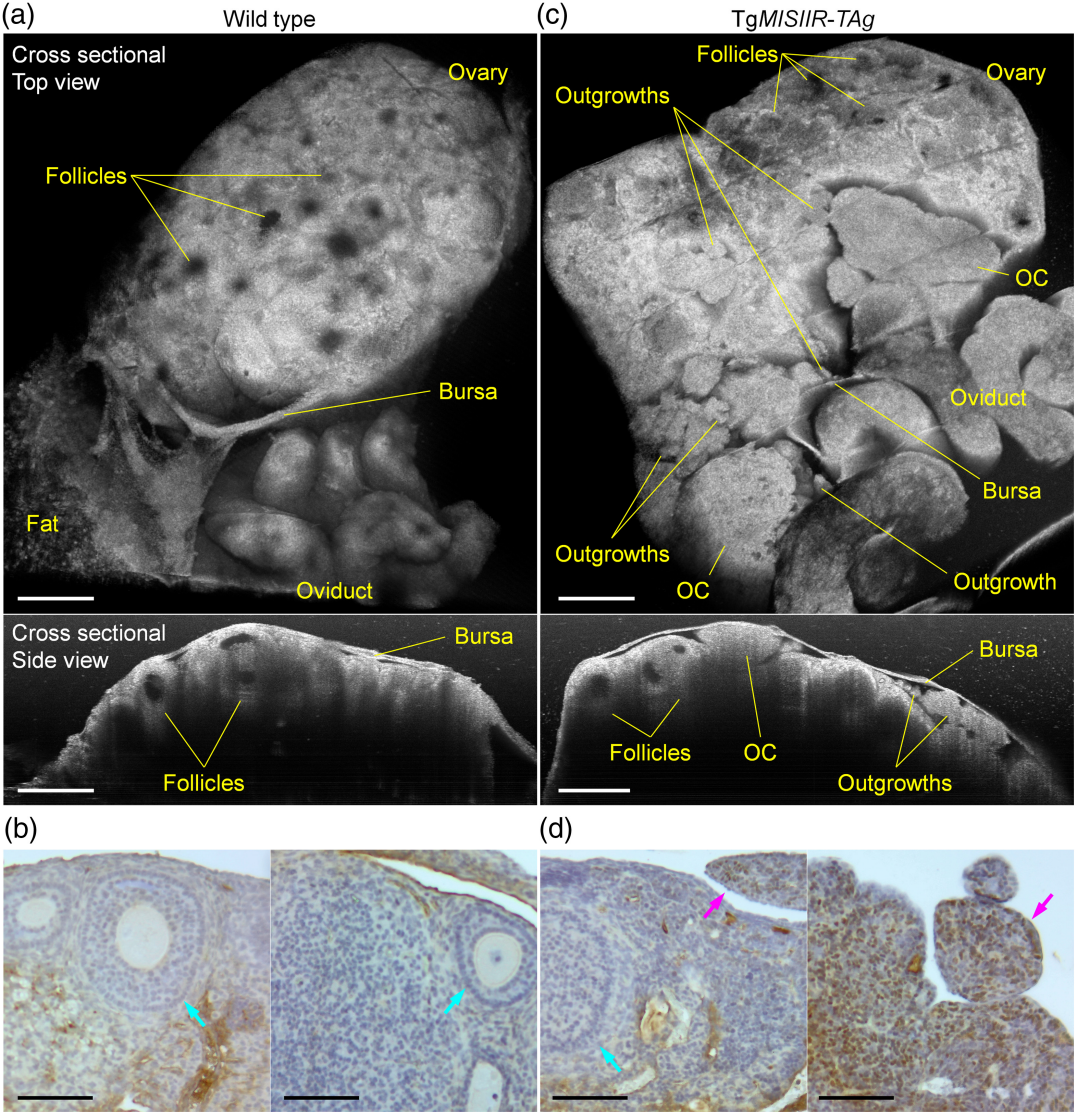
*In vivo* 3D imaging of the OC outgrowths from the Tg*MISIIR*-*TAg* transgenic mouse in comparison with the normal ovary in the wild-type mouse. (a) and (c) OCT images, including the top view (top) and side view (bottom), and (b) and (d) the related IHC staining of (a) and (b) the normal ovary and (c) and (d) the ovary with OC and its outgrowths. In the IHC images, brown nuclei indicate TAg-positive cells. Cyan arrows point at the follicles, and magenta arrows point at the OC outgrowths. Scale bars are 500  μm in OCT images and 100  μm in IHC images.

The *in vivo* OCT images of mouse ovaries with and without OC present distinct morphologies and structures. Figure 4(a) shows cross-sectional 3D visualizations of the normal ovary from a wild-type mouse, and the related IHC staining is shown in Fig. 4(b). From both the top view and the side view, the OCT images clearly reveal the follicles and their structures, similarly shown by Amaral et al.^30^ These include the circular structure of the follicle that represents layers of granulosa cells, the antrum that is filled with follicular fluid, and the oocyte within the antral follicle. The IHC images highlight the follicles and show no TAg-positive nuclei in the ovary. The normal follicles and their structures can also be seen in the ovary with OC, as shown from both OCT images in Fig. 4(c) and the IHC images in Fig. 4(d) from a DR26 mouse (age of 17.4 weeks). The OC tissues are mostly on top of the ovary, covering a portion of the ovary close to the oviduct, where the OC originates.^40^ Although there are large tumor masses, the relatively small OC outgrowths shown as spheroids can also be resolved in the OCT images. The smallest outgrowths in Fig. 4(c) were measured to have a size of ∼88  μm. Shown in Fig. 4, the ovarian bursa is a thin tissue layer that surrounds the ovary. The presence of OC and its outgrowths requires extra space and in many places appears to be compressed by the bursa. The results in Fig. 4 show the first high-resolution, large field of view, 3D image of the OC outgrowths *in vivo* in the mouse model, indicating their morphological and structural differences from the normal ovary.

**Fig. 5:**
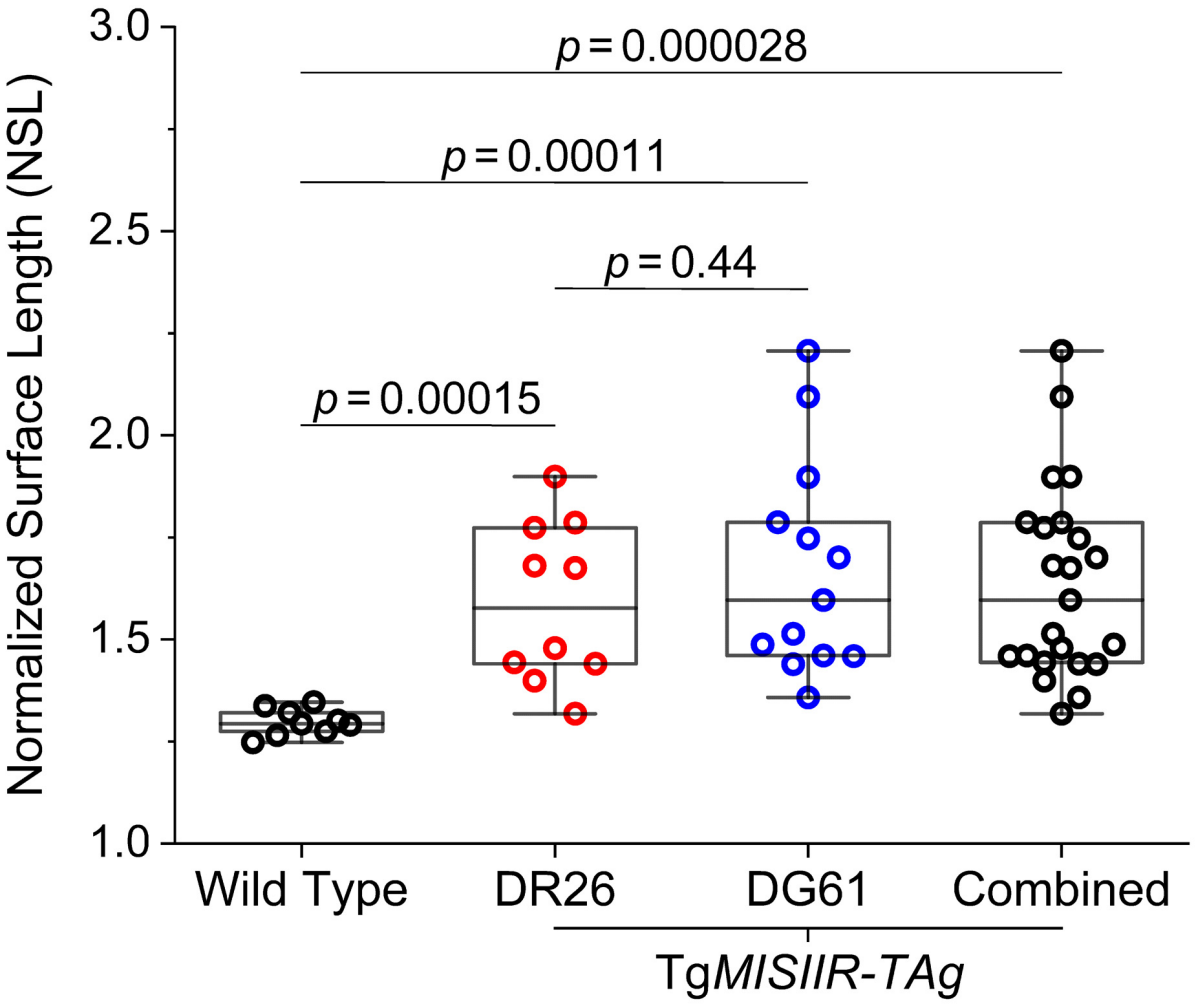
Comparison of the tissue surface morphologies with the NSL values between the OC outgrowths and the normal ovary. Box plots with median line and all data points. Whiskers are the minimum and maximum. All statistical tests are the Wilcoxon rank sum test (α=0.05).

Such a morphological difference of the OC outgrowth is further revealed quantitatively in Fig. 5 with the measurement of NSL. In comparison to the normal ovary in the wild-type mice, the OC outgrowths from both the DR26 mice and the DG61 mice have different NSL values, which are statistically significant (p values of 0.00015 and 0.00011, respectively). Notably, the NSL values of the OC outgrowths are not different between the DR26 mice and the DG61 mice (a p value of 0.44), indicating that the OC outgrowths from these two Tg*MISIIR*-*TAg* mouse lines can be grouped together for analysis. With data combined, the statistical test with a p value of 0.000028 demonstrates that the OC outgrowths present distinct surface morphologies in comparison to the normal ovary. Although all the wild-type mice were used for this analysis, only the Tg*MISIIR*-*TAg* mice that have apparent outgrowths on the ovary as shown in the OCT images were included in this quantitative assessment. These represent ∼67% of the DR26 mice and 52% of the DG61 mice.

**Fig. 6:**
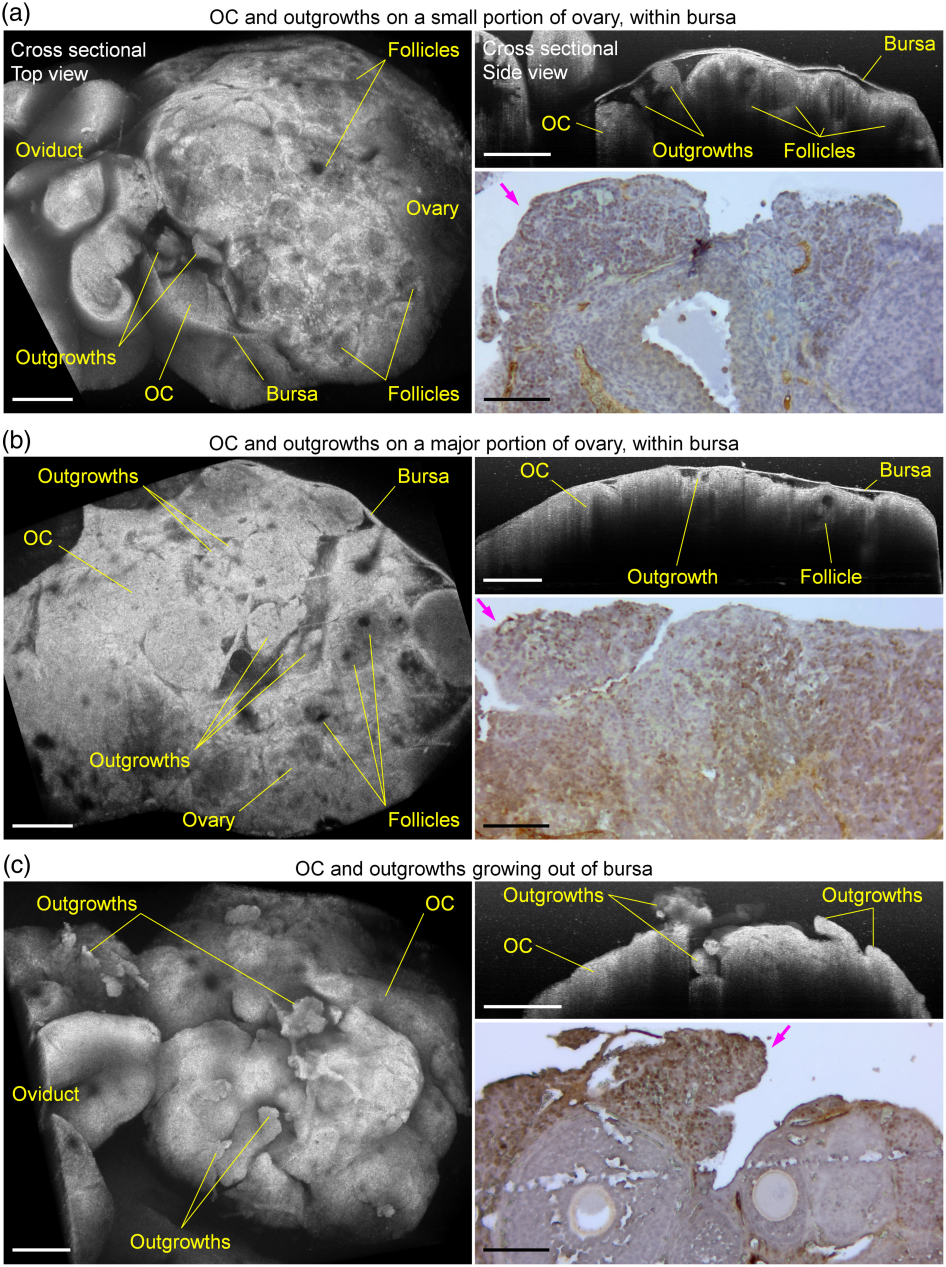
* In vivo* 3D OCT imaging of the OC outgrowths within and growing out of the ovarian bursa. OCT images, including the top view (left) and side view (right, top), and the related IHC staining (right, bottom) of the OC and its outgrowths (a and b) within the ovarian bursa covering (a) a small portion and (b) a major portion of the ovary, as well as (c) growing out of the bursa and extending into the peritoneal cavity. Magenta arrows point at the OC outgrowths. Scale bars are 500  μm in OCT images and 100  μm in IHC images.

To show the OC outgrowths within and growing out of the bursa, we selected three DR26 mice, and the cross-sectional 3D *in vivo* OCT images and the related IHC staining are shown in Fig. 6. These three mice have similar ages, which are 16.7 weeks in Fig. 6(a), 17.0 weeks in Fig. 6(b), and 16.0 weeks in Fig. 6(c). Figure 6(a) shows that the OC tissues are only present in the area close to the oviduct infundibulum, with most parts of the ovary having normal tissue structures, including the follicles, and both large tumors and small outgrowths can be observed from the images. In Fig. 6(b), the OC tissues cover more than half of the ovary, showing a big area of tumor masses as well as the outgrowths that appear as spheroids and are largely compressed by the bursa; however, the general follicular structures and oocytes can still be seen in the normal part of the ovary. Although the bursa appears to be stretched by the OC and its outgrowths, the entire tumor structures are enclosed within the bursa. Figure 6(c) shows that the OC outgrowths are outside of the bursa, with most of the bursa having disappeared. It is speculated that the significant growth of the tumor applies a sufficiently high stress onto the bursa to break it from the inside. Without the bursa, the outgrowths appear more robust, forming larger spheroids with branches of various shapes that further extend into the peritoneal cavity. Such outgrowths have distinct surface morphologies in comparison to the ones within the bursa, which were analyzed quantitatively with the NSL.

**Fig. 7:**
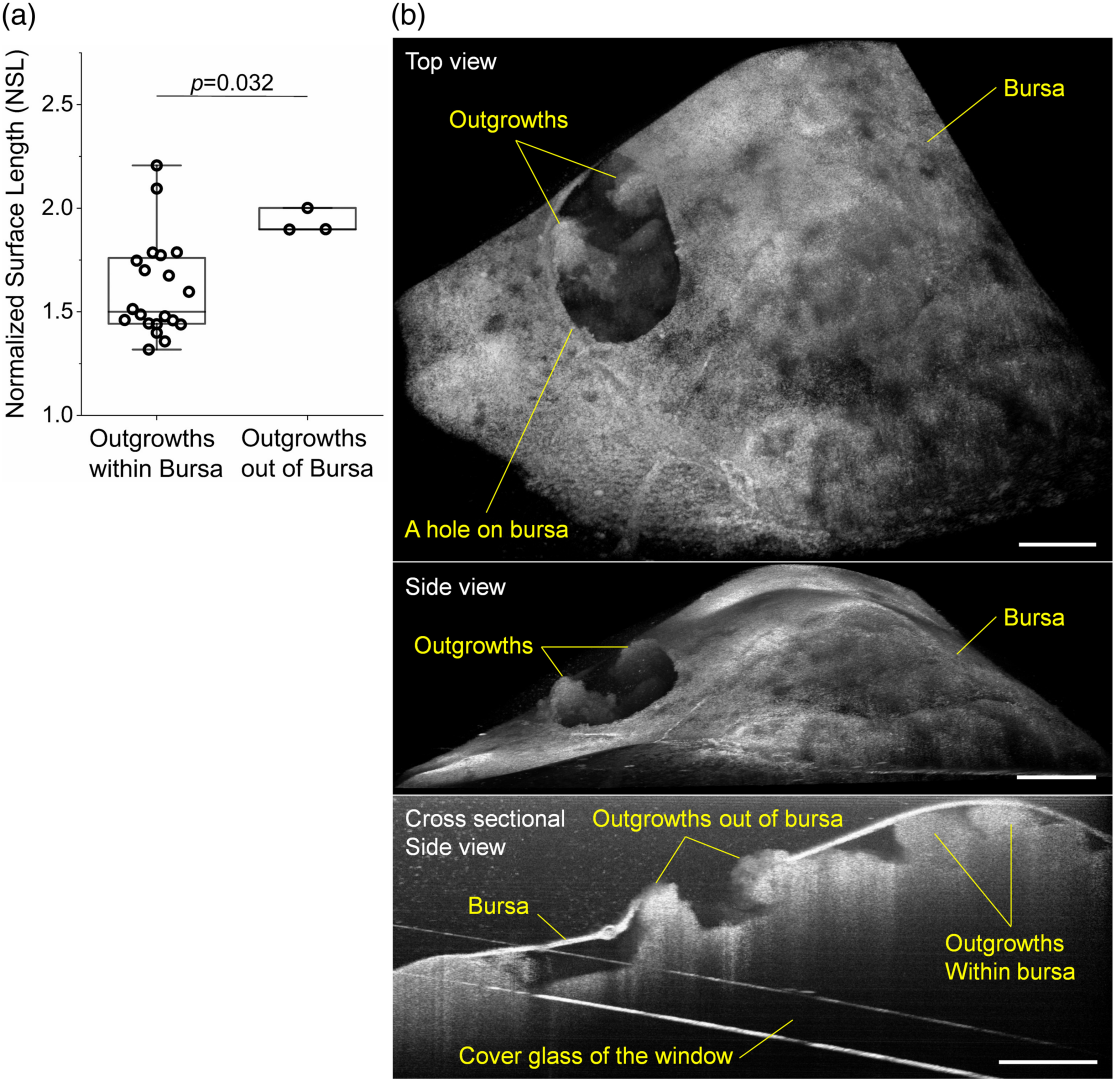
*In vivo* assessment and imaging of the OC outgrowths growing out of the ovarian bursa. (a) Comparison of the measured NSL values between outgrowths within and growing out of the bursa with the Wilcoxon rank sum test (α=0.05). Box plots with median line and all data points. Whiskers are the minimum and maximum. (b) OCT images, including the top view (top), side view (middle), and cross-sectional side view (bottom) showing the broken bursa with the OC outgrowths extending out of the bursa hole. Scale bars are 400  μm.

The OC outgrowths outside of the bursa present significantly larger NSL values than those inside the bursa (a p value of 0.032), as shown in Fig. 7(a), indicating that, without the spatial restriction from the bursa, the OC outgrowths are able to develop more complex structures. The three mice in Fig. 7(a) with outgrowths growing out of the bursa include two DR26 mice (ages of 12.7 and 16.0 weeks) and one DG61 mouse (age of 60.7 weeks). Although the DG61 mouse is older than most of the other DG61 mice that have OC outgrowths within the bursa, the ages of the two DR26 mice are below the average of the other DR26 mice that have OC outgrowths within the bursa. It is currently unknown how the bursa from these mice disappears or breaks, but the OCT image data from one DR26 mouse (age of 12.7 weeks) show that the bursa was partially broken, suggesting a relatively slow or stepwise process. Specifically, Fig. 7(b) shows the hole in the bursa, where two OC outgrowths have their tips out of the bursa hole. Based on this, it is our speculation that, with more outgrowths becoming larger, the bursa hole can become bigger, eventually exposing most of the outgrowths directly to the peritoneal cavity.

**Fig. 8:**
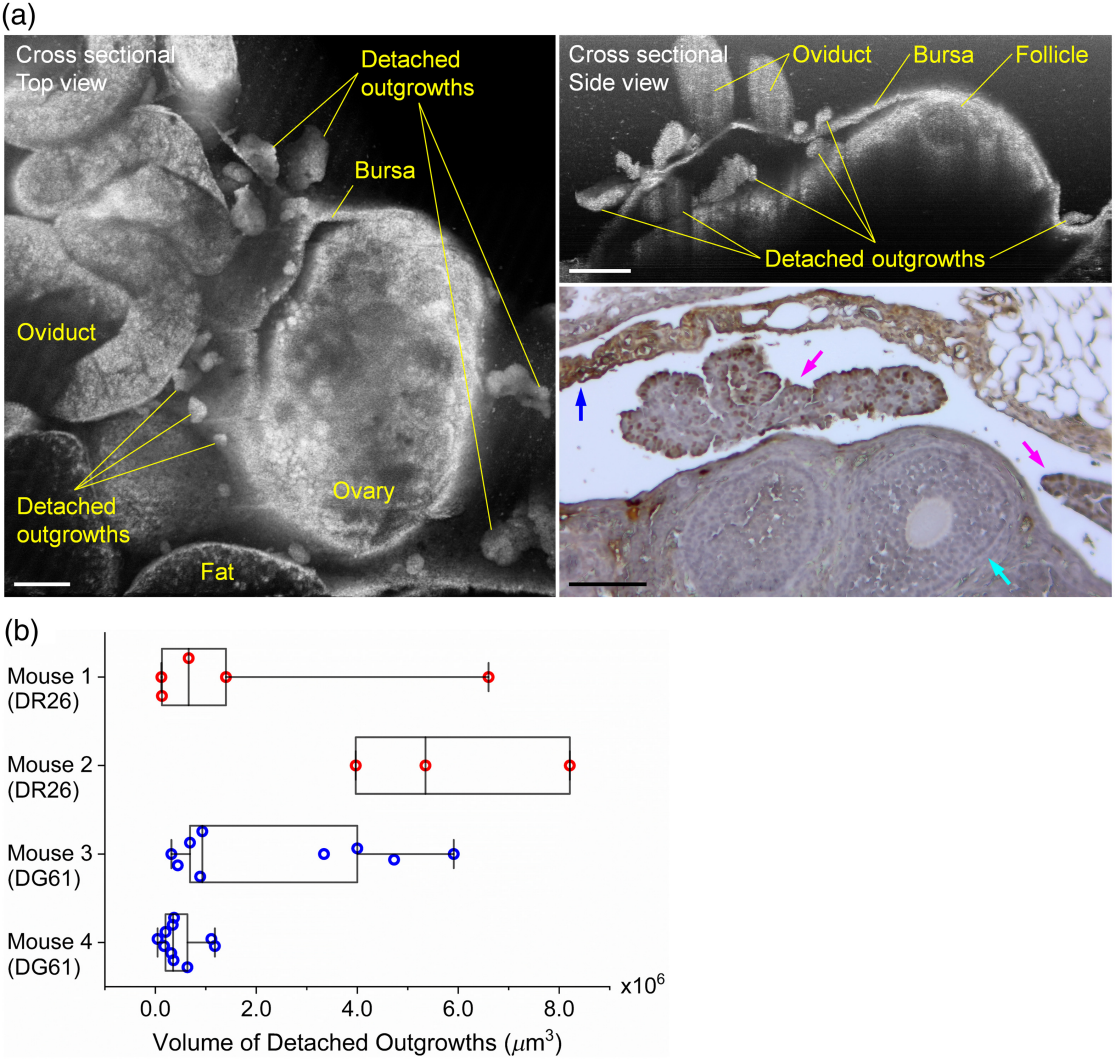
*In vivo* 3D OCT imaging of the detached OC outgrowths within and outside of the bursa. (a) OCT images, including the top view (left) and side view (right, top), and the related IHC staining (right, bottom) of the detached OC outgrowths. In the IHC staining, brown nuclei indicate TAg-positive cells. Cyan arrow points at a follicle, magenta arrows point at the detached outgrowths, and blue arrow points at the bursa. Scale bars are 300  μm in OCT images and 100  μm in IHC image. (b) The measured volumes of the detached OC outgrowths from four mice (two DR26 mice and two DG61 mice) showing the heterogeneity in size. Each data point is from one detached outgrowth. Box plots with median line and all data points. Whiskers are the minimum and maximum.

The *in vivo* imaging and assessment of detached OC outgrowths are shown in Fig. 8. Revealed by the cross-sectional 3D OCT images in Fig. 8(a) from a DG61 mouse (age of 34.0 weeks), the detached outgrowths appear as spheroids of various shapes and sizes and are located both inside and outside of the ovarian bursa. The IHC staining confirms the presence of TAg-positive cells in the detached outgrowths. Based on the 3D OCT images, volume measurements shown in Fig. 8(b) quantitatively confirm the heterogeneity in size of the detached outgrowths. Two DR26 mice and two DG61 mice were used in this analysis with their ages of 17.4 weeks (mouse 1), 17.4 weeks (mouse 2), 34.0 weeks (mouse 3), and 39.9 weeks (mouse 4).

**Fig. 9:**
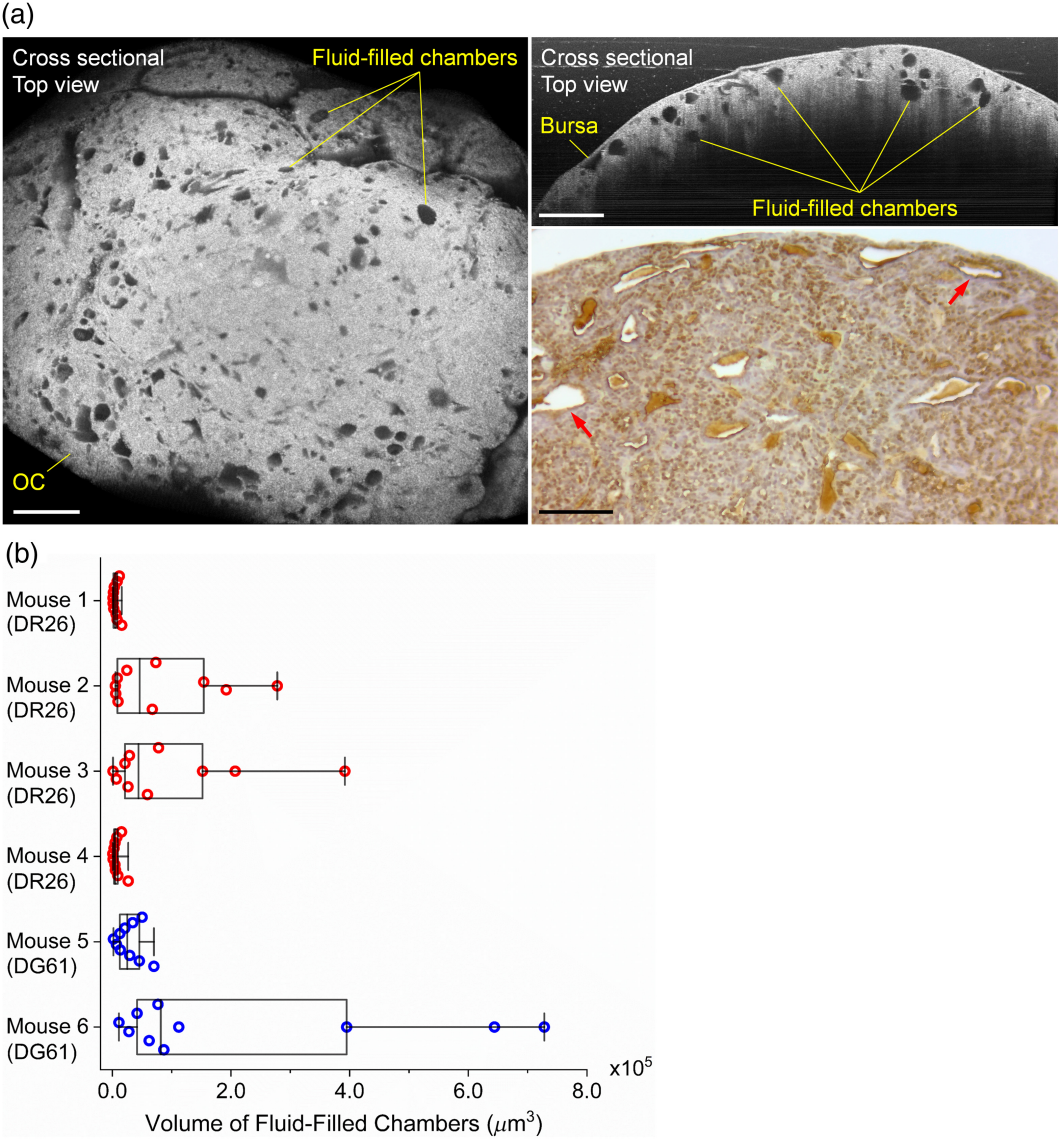
*In vivo* 3D OCT imaging of the fluid-filled chambers inside the OC tissue. (a) OCT images, including the top view (left) and side view (right, top), and the related IHC staining (right, bottom) of the OC tissue with the fluid-filled chambers. In the IHC staining, brown nuclei indicate TAg-positive cells. Red arrows point at the fluid-filled chambers. Scale bars are 500  μm in OCT images and 100  μm in IHC image. (b) The measured volumes of the fluid-filled chambers from six mice (four DR26 mice and two DG61 mice) showing the heterogeneity in the chamber size within and across tumors. Each data point is from one chamber. Box plots with median line and all data points. Whiskers are the minimum and maximum.

Fluid-filled chambers were found inside the OC tissues during our imaging experiments, and Fig. 9 presents the *in vivo* visualization and assessment of such chambers in the tumor. The cross-sectional 3D OCT images in Fig. 9(a) from a DR26 mouse (age of 17.3 weeks) show a wide distribution as well as a large variety of sizes and shapes of these chambers. They are located at different depths of the OC tissue, which is confirmed by IHC staining. From 3D views, some of the chambers appear connected, and a detailed inspection shows small particles of approximately cell size floating inside the chamber or attaching to the chamber wall. The measurements of the chamber volume are shown in Fig. 9(b), and it shows that the size heterogeneity not only exists within the OC tissue from one mouse but also presents across mice. Four DR26 mice and two DG61 mice were used in this analysis with their ages of 17.4 weeks (mouse 1), 16.9 weeks (mouse 2), 17.3 weeks (mouse 3), 16.0 weeks (mouse 4), 60.7 weeks (mouse 5), and 35.4 weeks (mouse 6).

## 4 Discussion

This work achieves the first *in vivo* 3D imaging of the OC outgrowths in the mouse model; the image quality for visualizing and analyzing the OC and its outgrowths is unprecedented. This provides a range of new possibilities to study the OC dissemination *in vivo*, especially the dynamics underlying the key processes.

Although the OC outgrowths can be directly visualized and distinguished from the structure OCT images, it is challenging to segment these outgrowths and the OC tissues from the ovary only based on the structure OCT image. Additional contrast is needed to enable such segmentations. It is expected that the OC cells, the normal cells, and the extracellular matrix in the ovary have different dynamics at the submicron level, and thus, dynamic OCT^25^^,^^27^ could potentially be used to provide further analysis and assist the tissue segmentation. Further image processing and learning-based approaches as shown previously^31^^,^^32^ can also be useful for efficient segmentation of targeted tissue structures.

In this work, the OCT-imaging location on the ovary is marked for sectioning and IHC staining. However, this process does not produce accurate correspondence between the OCT image and the IHC image, which is a general challenge. Recently, a micro-registration method between OCT and histology has been developed,^48^ and similar approaches could be potentially useful for OCT imaging of the OC outgrowths, which is important for further analysis of the OCT images.

For advanced strategies to reduce OC outgrowth dissemination, it is imperative to understand the factors causing the outgrowths to detach from the primary site. The imaging approach shown here can potentially provide efficient *in vivo* evaluation of the effects of both chemical cues (e.g., chemotherapy) and mechanical cues (e.g., fluid flow) on the outgrowth detachment, which is the focus of our future work. However, it is worth noting that the insights from such studies are specific to the transgenic mouse model, and the relevance to the spread of human OC requires further investigation. In this study, the results on the various sizes of the detached OC outgrowths imply that the outgrowth size might not play a role in the detachment. This is supported to some extent by the previous work showing that both individual OC cells and multicellular OC cell clusters can detach.^7^

Our results reveal the fluid-filled chambers inside the OC tissue, and we have visualized cell-sized particles in the chambers. It is currently not clear how these chambers form and what such particles are, though we speculate that inflammation inside the tumor generates the fluid-filled space and that macrophages or tumor cells within the fluid are captured in the 3D OCT image. The chambers appear heterogeneous, which might be affected by the stage of inflammation. It is known that inflammation plays a role in tumor progression and can promote the spread of OC,^49^ and our imaging result suggests an *in vivo* approach to potentially probe this process. For analyzing the fluid, OCT-guided micropipette fluid extraction, which is similar to the setup for microinjection,^50^ could be used. In addition, *in vivo* OCT imaging can present a robust imaging guidance to manipulate the tissue and fluid environment of the OC outgrowths for studying their behaviors in response to the change of their environment *in vivo*. For example, a recently established OCT-guided bioprinting approach^51^ can be useful for such potential studies.

The OC outgrowths form and detach over days, with their structural changes visible over hours.^8^ OCT has the suitable imaging speed to capture such dynamics and also has the functional imaging capabilities to extract additional tissue and cell properties.^22^^,^^52^ Nevertheless, due to its typical imaging scales and the basic structure imaging contrasts, OCT is largely limited to tissue-level imaging, and multimodality imaging based on this* in vivo* approach, such as adding multiphoton microscopy,^53^ photoacoustic microscopy,^54^ or Brillouin microscopy,^55^ is promising to integrate a wider range of molecular, cellular, and metabolic information to elucidate the OC dissemination process. The intravital window has been previously demonstrated for prolonged and longitudinal imaging over multiple hours and a few days;^42^^,^^56^ however, for repeated imaging analysis over a longer period, such as weeks, to monitor and evaluate the OC outgrowth, further feasibility studies with a refinement of the window design are needed.

From the technical aspect of OCT, achieving a deeper depth of view into the OC tissue is largely restricted by the significant sensitivity roll-off from the spectral domain system in this study. Using a swept source OCT system promises to extend the imaging depth and acquire more structural information deeper inside the tumor. Traditionally, swept source OCT has an inferior spatial resolution to spectral domain systems primarily due to the limitations in the available wavelengths; however, advancements in the swept laser source have demonstrated a comparable spatial resolution,^57^ pointing to the potential use of swept source OCT for even better 3D imaging quality of the OC outgrowths *in vivo*.

## 5 Conclusion

We report the first *in vivo* high-resolution 3D imaging of the OC outgrowths in the mouse model with OCT and an intravital window. The 3D OCT image reveals the detailed morphology and structures of the OC and its outgrowths in the Tg*MISIIR*-*TAg* transgenic mice, which show clear differences from the normal ovary in the wild-type mouse. Such differences are quantitatively analyzed, which demonstrates statistical significance. We present the various OC outgrowths within and growing out of the ovarian bursa and show that the outgrowths outside of the bursa have more complex surface morphologies than those inside the bursa. We further present 3D images of the detached OC outgrowths within and outside of the bursa and the fluid-filled chambers formed inside the tumor, demonstrating their heterogeneity in size with volume measurements. This work paves the way for understanding the high-resolution dynamics of OC dissemination *in vivo*.

## Data Availability

This work did not generate codes that are beyond the generally used functions in MATLAB and ImageJ macros. The data that support the findings in this work are available upon request to the corresponding author.
